# Supplementary material for: Safety and efficacy of the mRNA BNT162b2 vaccine against SARS-CoV-2 in five groups of immunocompromised patients and healthy controls in a prospective open-label clinical trial
Source: eBioMedicine. 2021 Nov 30;74:103705. doi: 10.1016/j.ebiom.2021.103705 (PMC8629680; doi:10.1016/j.ebiom.2021.103705)

**Supplementary Figure 1.** Seroconversion and antibody titres in all patients according to intention to treat (ITT). a) SARS-CoV-2 specific antibody titres ≥ 0.8 U/ml for each time point in the five immunocompromised groups and control group. b) Dynamics of SARS-CoV-2 specific antibody titres for all patients who received at least dose 1. c) Dynamics of SARS-CoV-2 specific antibody titres for healthy controls and all patients in each respective group who received at least dose 1. Seroconversion in patients before receiving dose 1 (red), in patients who had not received dose 2 (blue), or where baseline samples or day 35 samples were missing.


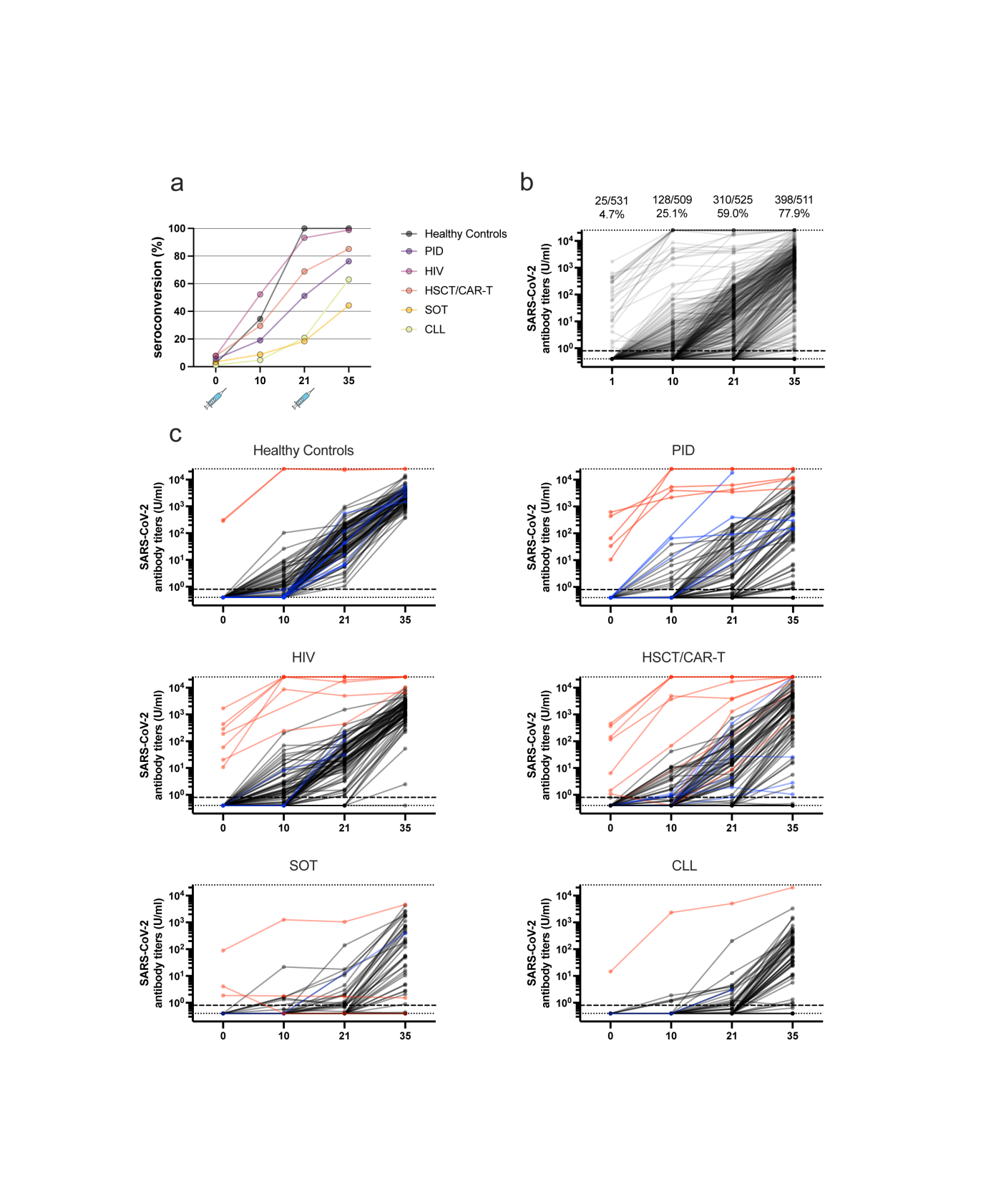

Supplement: Supplementary file 1 [file mmc1.docx]
